# A Prospective Study on Risk Factors for Acute Kidney Injury and All-Cause Mortality in Hospitalized COVID-19 Patients From Tehran (Iran)

**DOI:** 10.3389/fimmu.2022.874426

**Published:** 2022-07-08

**Authors:** Zohreh Rostami, Giuseppe Mastrangelo, Behzad Einollahi, Eghlim Nemati, Sepehr Shafiee, Mehrdad Ebrahimi, Mohammad Javanbakht, Seyed Hassan Saadat, Manouchehr Amini, Zahra Einollahi, Bentolhoda Beyram, Luca Cegolon

**Affiliations:** ^1^Nephrology and Urology Research Center, Baqiyatallah University of Medical Sciences, Tehran, Iran; ^2^Department of Cardiac, Thoracic, Vascular Sciences and Public Health, Padua University, Padua, Italy; ^3^School of Medicine, Shahid Beshest University of Medical Sciences, Tehran, Iran; ^4^Nephrology Research Center, Shariati Hospital, Tehran University of Medical Sciences, Tehran, Iran; ^5^Scholl of Medicine, Tehran University of Medical Sciences, Tehran, Iran; ^6^Department of Medical, Surgical and Health Sciences, University of Trieste, Trieste, Italy; ^7^Public Health Department, University Health Agency Giuliano-Isontina (ASUGI), Trieste, Italy

**Keywords:** acute kidney injury, COVID-19, electrolyte abnormalities, renal failure, SARS-CoV-2

## Abstract

**Background:**

Several reports suggested that acute kidney injury (AKI) is a relatively common occurrence in hospitalized COVID-19 patients, but its prevalence is inconsistently reported across different populations. Moreover, it is unknown whether AKI results from a direct infection of the kidney by SARS-CoV-2 or it is a consequence of the physiologic disturbances and therapies used to treat COVID-19. We aimed to estimate the prevalence of AKI since it varies by geographical settings, time periods, and populations studied and to investigate whether clinical information and laboratory findings collected at hospital admission might influence AKI incidence (and mortality) in a particular point in time during hospitalization for COVID-19.

**Methods:**

Herein we conducted a prospective longitudinal study investigating the prevalence of AKI and associated factors in 997 COVID-19 patients admitted to the Baqiyatallah general hospital of Tehran (Iran), collecting both clinical information and several dates (of: birth; hospital admission; AKI onset; ICU admission; hospital discharge; death). In order to examine how the clinical factors influenced AKI incidence and all-cause mortality during hospitalization, survival analysis using the Cox proportional-hazard models was adopted. Two separate multiple Cox regression models were fitted for each outcome (AKI and death).

**Results:**

In this group of hospitalized COVID-19 patients, the prevalence of AKI was 28.5% and the mortality rate was 19.3%. AKI incidence was significantly enhanced by diabetes, hyperkalemia, higher levels of WBC count, and blood urea nitrogen (BUN). COVID-19 patients more likely to die over the course of their hospitalization were those presenting a joint association between ICU admission with either severe COVID-19 or even mild/moderate COVID-19, hypokalemia, and higher levels of BUN, WBC, and LDH measured at hospital admission. Diabetes and comorbidities did not increase the mortality risk among these hospitalized COVID-19 patients.

**Conclusions:**

Since the majority of patients developed AKI after ICU referral and 40% of them were admitted to ICU within 2 days since hospital admission, these patients may have been already in critical clinical conditions at admission, despite being affected by a mild/moderate form of COVID-19, suggesting the need of early monitoring of these patients for the onset of eventual systemic complications.

## Background

SARS-CoV-2 is known for its ability to invade various organs (1). Earlier studies on the impact of COVID-19 focused on the pulmonary system, and dysfunctions of other organs were attributed to hyper-inflammatory response and thrombophilia-inducing multiorgan failure (MOF).

ACE-2 and TMPRSS-2, surface cell proteins expressed by various tissues, are targeted by SARS-CoV-2. In addition to the respiratory system, ACE-2 and TMPRSS-2 are also expressed in the gastrointestinal tract, brain, and vessels (2–5). Furthermore, ACE-2 is highly expressed in renal proximal tubules, where SARS-CoV-2 particles were detected postmortem in podocytes of COVID-19 patients, suggesting that the kidneys could also be one of the targets of SARS-CoV-2 (6, 7). Acute kidney injury (AKI)—a common finding in hospitalized COVID-19 patients—can interfere with the conventional management of COVID-19, resulting in poorer prognosis in terms of higher risk of mortality, intensive care unit (ICU) admission, and prolonged hospitalization (8, 9). It is unknown, however, whether AKI results from a direct infection of the kidney by SARS-CoV-2 or as a consequence of the physiologic disturbances and therapies used to treat the viral disease (10).

Up until November 14, 2021, the cumulative number of COVID-19 infections globally was 3,800,000,000, with a 1%–2% hospitalization rate and a mortality rate of 194.5/100,000 (11). Prevalence of AKI in COVID-19 patients is inconsistently reported, ranging from 0.5% in China (12) to 80% among critically ill COVID-19 patients in France (13, 14). While AKI prevalence among COVID-19 patients was low in initial reports from China, subsequent figures became much higher, suggesting the kidney as one of the main organs targeted by SARS-CoV-2 (15). For instance, in an observational cohort study conducted in a large tertiary care university hospital in Wuhan (China), enrolling all consecutive COVID-19 inpatients older than 65 years during January 2020, the prevalence of AKI was 14% (16). In a meta-analysis on 6,945 COVID-19 patients from China, Italy, the UK, and the USA recruited from 2019 to May 11, 2020, the incidence of AKI was 8.9% [95% CI: 4.6–14.5] (17). Higher AKI figures (46%) were observed among 3,993 COVID-19 patients aged ≥18 years admitted to the Mount Sinai Health System (New York) from February 27 to May 30, 2020 (18), and in another study (rate of 32%) in New York city on a cohort of 5,216 US veterans hospitalized for COVID-19 from February 1, 2020, to July 23, 2020 (16, 19). Likewise, the incidence of AKI on 5.449 COVID-19 patients admitted to 18 university and community hospitals of New York between March 1 and April 5, 2020, was 36.6% (20). Lower AKI figures have been reported in Europe during the first pandemic wave, for instance in a multicenter study on 1,855 admissions for COVID-19 in London hospitals from January 1, 2020, up to May 14, 2020, where 455 patients (a rate of 24.5%) developed AKI (21). Likewise, prevalence of AKI among hospitalized COVID-19 patients was estimated to be 22.4% in an Italian study (22). By contrast, a study from Brazil reported an incidence of AKI of 71% among critically ill COVID-19 patients (23).

In view of the above, we carried out a prospective study on hospitalized COVID-19 patients in Tehran (Iran) with a double aim:

- To estimate the prevalence of AKI, since it varies by geographical settings, time periods, and populations studied; and
- To investigate the risk factors predicting AKI occurrence, assessing their impact on survival of hospitalized COVID-19 patients.

## Methods

**Figure 1:**
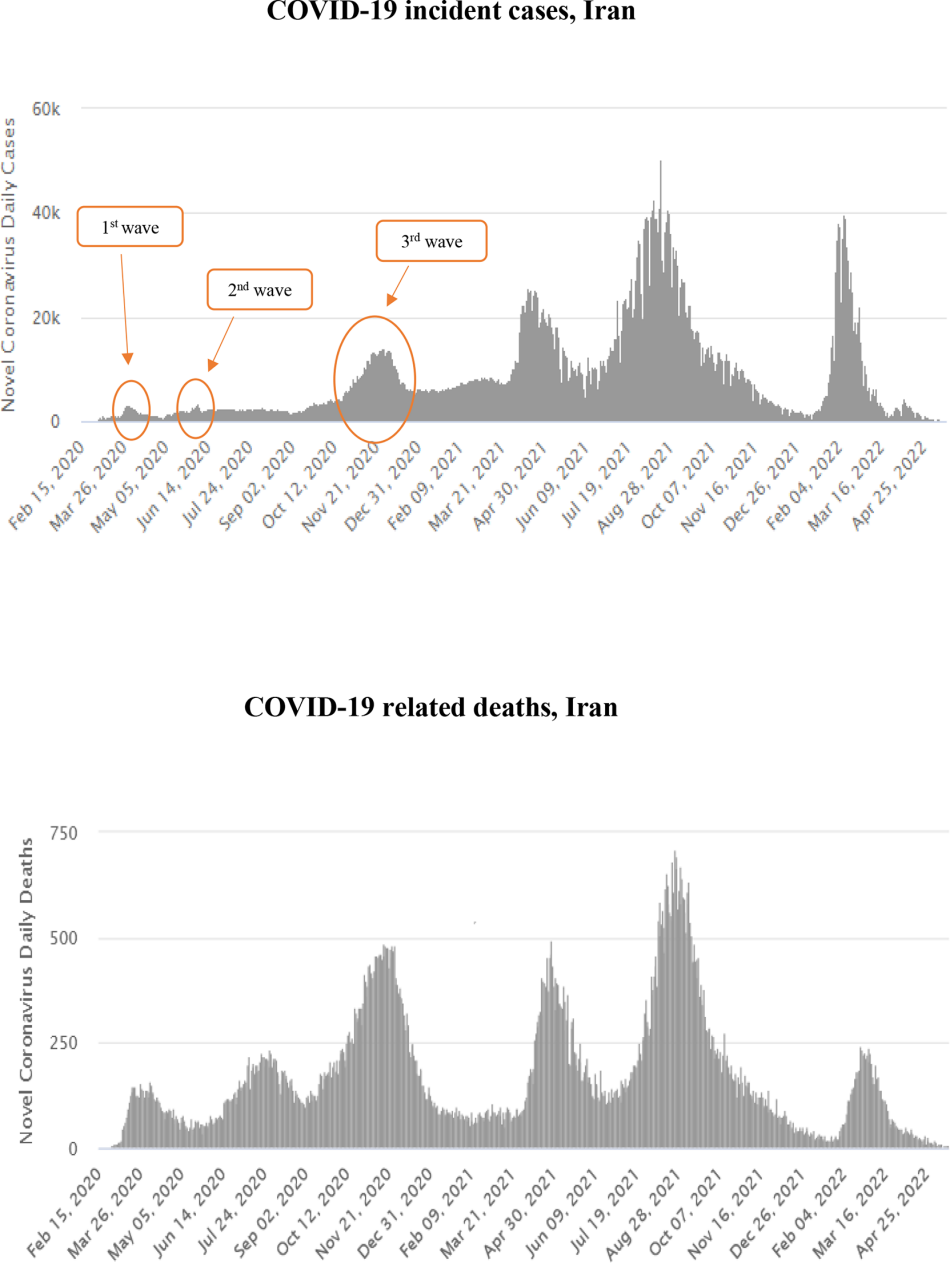
Temporal distribution of COVID-19 incident cases (upper panel) and deaths (lower panel) in Iran, February 2020–April 2022. (24)

This single-center longitudinal prospective study was conducted at Baqiyatallah general hospital in Tehran (Iran), from October 22, 2020, until January 7, 2021, during the third wave of the COVID-19 pandemic (**Figure 1**) (24). The study received approval from the research ethic committee of Baqiyatallah University of Medical Sciences. COVID-19 diagnosis was confirmed by RT-PCR on nasopharyngeal swabs, as per WHO guidelines (25).

**Figure 2:**
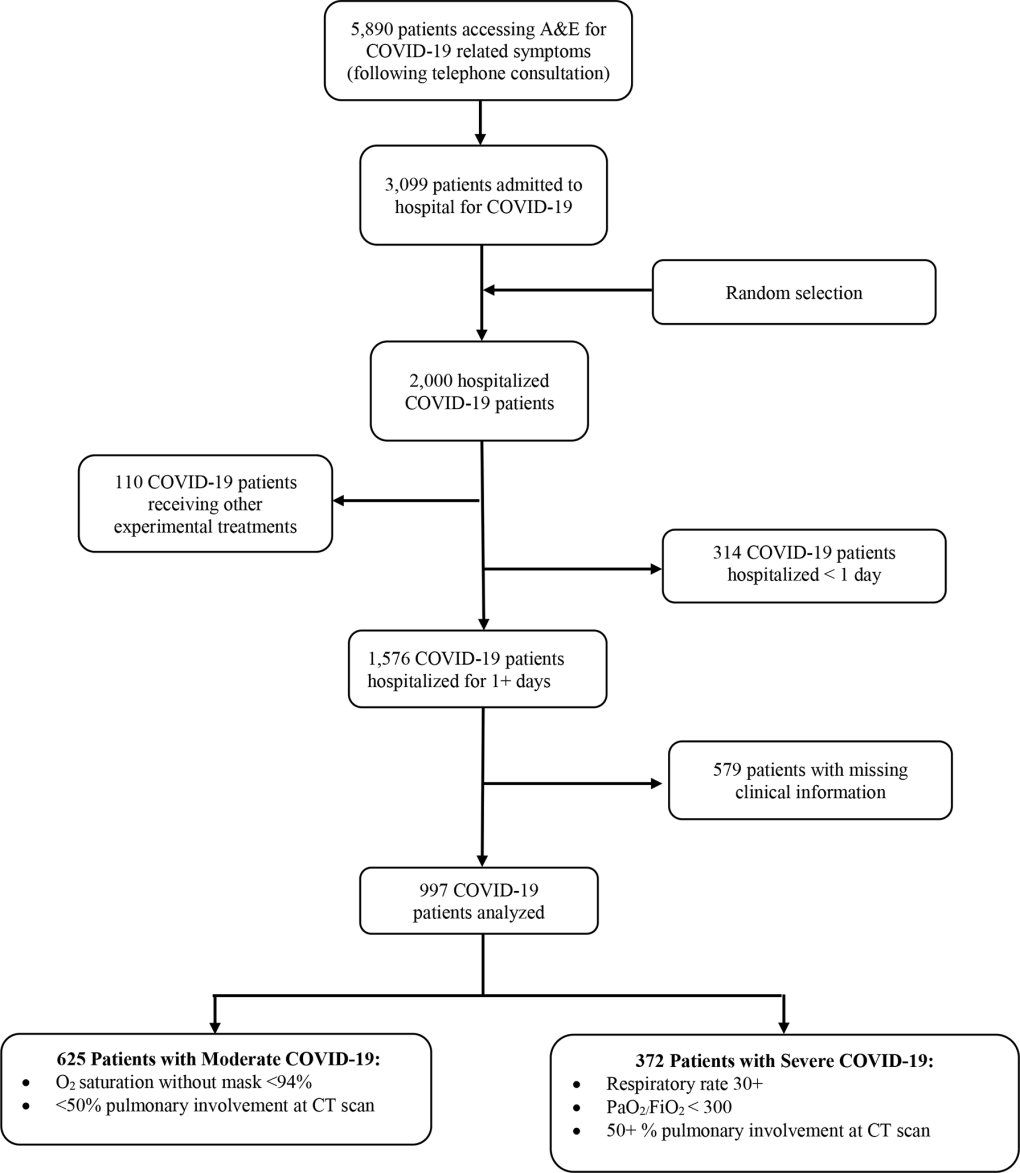
Flowchart with inclusion/exclusion criteria of hospitalized COVID-19 patients enrolled in this study from October 22, 2020–January 7, 2021.

Following triage telephone consultations, 5,890 patients with COVID-19 symptoms were referred to Accident & Emergency (A&E) of Baqiyatallah general hospital of Tehran (Iran) from October 22, 2020, until January 7, 2021. Two thousand COVID-19 patients were randomly selected (using a simple random code generator software) among those 3,099 hospitalized for more than 1 day. Patients receiving alternative experimental treatments (N = 110) were excluded since they were part of other clinical trials. Furthermore, patients with missing data on past medical history and serum creatinine and those with only one documented creatinine measurement were excluded (**Figure 2**). The final number of patients analyzed in this study was 997, broken down into 625 patients affected by mild/moderate COVID-19 and 372 patients with severe disease (**Figure 2**).

### Variables

All variables were collected from hospital records (and stratified as follows).

* Oxygen supplement (strata: mask, mask with bag reserve, non-invasive ventilation, intubation);

* Degree of severity of COVID-19.

⚬ Mild COVID-19 (26):

a) Any signs and symptoms of COVID-19 (fever, cough, sore throat, malaise, headache, muscle pain, nausea, vomiting, diarrhea, loss of taste and smell), BUT

b) No shortness of breath, dyspnea, or abnormal chest imaging.

⚬ Moderate COVID-19 (26):

a) ≥94% O_2_ saturation without mask, AND

b) <50% lung involvement at imaging.

⚬ Severe COVID-19 (26):

a) SpO_2_ <94%; OR

b) Respiratory rate ≥30, OR

c) PaO_2_/FiO_2_ <300 mmHg; OR

d) Signs of pulmonary involvement at imaging ≥50%.

* Sex (female and male);

* Age (years: mean ± SD; classes: <47; 47–56; 57–65; 66+ years);

* Timeline of AKI onset (days since hospital admission: mean ± SD; classes: <2; 2–3; 4–6; and 7+ days);

* Length of hospital stay (LoS: mean ± SD; classes: <7; 7–9; 10–13; 14+ days);

* ICU admission (no, yes)

* Timeline of ICU (days since hospital admission: mean ± SD; classes: <2; 3–5; 6+ days);

* Mortality (no, yes);

* Comorbidities: diabetes mellitus (no, yes); hypertension (no, yes); ischemic heart disease (IHD: no, yes); chronic heart failure (CHF: no, yes); end-stage liver disease (ESLD: no, yes); chronic obstructive pulmonary disease—interstitial lung disease (COPD-ILD: no, yes); chronic kidney disease (CKD: no, yes if eGFRr <45); [CKD: no, yes if Estimated Glomerular Filtration Rate (eGFR) <45]

* AKI stage (stage 1, stage 2, stage 3).

**Figure 3:**
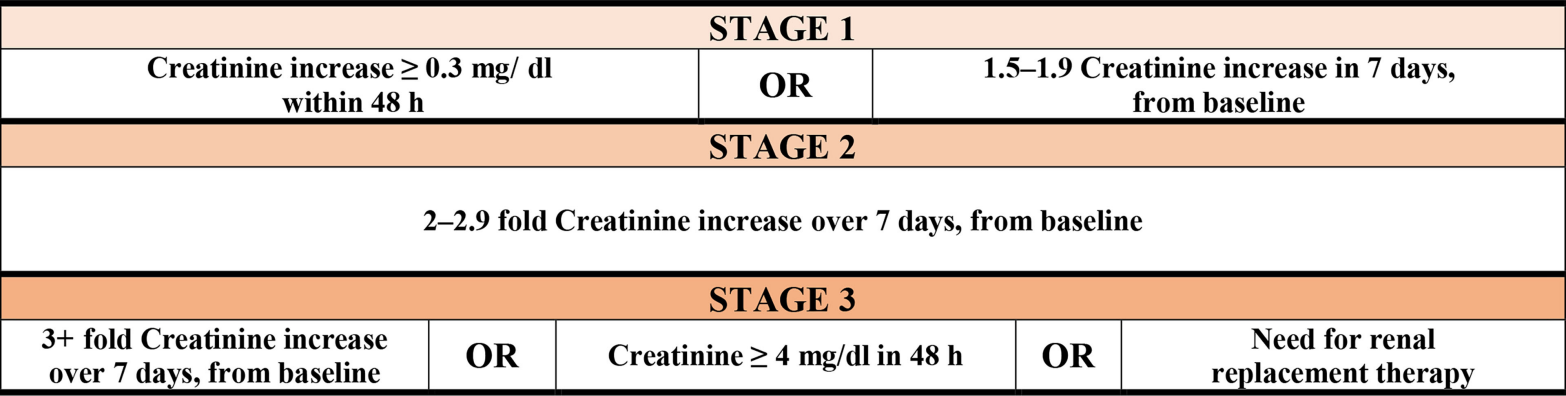
Stages of renal failure by kidney disease according to Improving Global Outcomes (KDIGO) guidelines.

The International Classification of Diseases (ICD10) was used to evaluate mortality and comorbidities. AKI and stage of renal failure were identified using Kidney Disease: Improving Global Outcomes (KDIGO) guidelines (27) (**Figure 3**).

* eGFR (mL/min/1.73 m^2^): mean ± SD. The following formula for modification of diet in renal disease (MDRD) was used to estimate the glomerular filtration rate:

**eGFR = 186 × (Cr)^−1.154^ × (Age)^−0.203^× (1.212 if Black) × (0.742 if Female)**

* Electrolyte imbalance: natremia (hypo Na^+^ <135 mEq/l; Hyper Na^+^ >145 mEq/l); kalemia (Hypo K^+^ <3.5 mEq/l; hyper K^+^ > 5 mEq/l); magnesemia (hypo Mg^2+^ <1.46 mg/dl; hyper Mg^2+^ >2.68 mg/dl); calcemia (hypo Ca^2+^ <8.8 mg/dl; hyper Ca^2+^ >10.5 mg/dl).

* White blood cells, WBC (× 10^9^/l): mean ± SD;

* Platelets (× 10^9^/l): mean ± SD;

* Hemoglobin (g/dl): mean ± SD;

* Lactate de-hydrogenase, LDH (U/L); mean ± SD;

* Blood creatinine (mg/dL): mean ± SD;

* Blood urea nitrogen (BUN; mg/dL): mean ± SD.

All clinical information but ICU referral was collected at hospital admission. Blood creatinine and BUN were also measured at end of follow-up (hospital discharge or death).

The national guidelines of the Iran Ministry of Health (MoH) and medical education as well as WHO guidelines on management of COVID-19 (26, 28–30) were followed for the hospital management of COVID-19 patients and decision making on ICU admissions.

After the initial admission, all patients were evaluated, monitored, and treated for volume depletion and high blood sugar. All patients were stabilized in A&E department before being transferred to COVID-19 wards and constantly monitored for their hemodynamic status during hospitalization. Treatment of patients was mainly supportive and based on WHO guidelines on COVID-19 patient management at the time of the study. The only non-steroid-anti-inflammatory drug (NSAID) used was naproxen, administered routinely but to patients with low eGFR and with other contradictions. Enoxaparin or heparin was used as anticoagulants. In patients with mild/moderate COVID-19, dexamethasone was administered, whereas in those affected by severe disease methylprednisolone pulse was employed. Antibiotic therapy was administered only in case of secondary bacterial infection.

Treatment drugs were adjusted based upon eGFR and administration of diuretics; ACE inhibitors and angiotensin II receptor blockers were refrained in patients at risk of AKI. Furthermore, remdesivir was not administered in patients with eGFR<30 mg/dl/1.73 m^2^, because of its debated nephrotoxicity risk (31).

### Statistical Analysis

Distribution of variables by AKI status (yes vs. no) was estimated by chi-squared test in case of categorical terms, whereas ANOVA was employed for comparison of continuous terms by AKI. The distribution of timeline of ICU referral since hospital admission was contrasted by the mean length of hospital stay (LoS, in days), AKI onset (yes vs. no), timeline of AKI onset (days since hospital admission), and vital status at end of follow-up (death or discharge). The mean and median concentration of BUN and blood creatinine were contrasted between hospital admission and end of hospital dischargefollow-up by Wilcoxon test.

This prospective study was conducted over a short period of time collecting both clinical information and several dates (of: birth; hospital admission; AKI onset; ICU admission; hospital discharge; death). In order to examine how the above risk factors influenced mortality risk and AKI incidence during hospitalization in a particular point in time, survival analysis using the Cox proportional-hazard models was adopted. Two separate multiple Cox regression models were fitted for each outcome (AKI and death). The two multivariable models were built up only including variables significant at univariable analysis.

Statistical interaction was modeled by including a product of ICU and severity of COVID-19 in the regression model to evaluate whether COVID-19 severity modified the association between ICU and in-hospital mortality. Similarly, we assessed the interaction between ICU and AKI and between COVID-19 severity and AKI. Results were expressed as hazard ratio (HR) with 95% confidence interval (95% CI). Non-significant terms at multivariable analysis were omitted from the respective tables. The level of significance for each test was set at 0.05. All terms and interactions not being significant were dropped out, and the corresponding results were not shown in the tables.

Statistical analysis was conducted using Stata 14.2 (Stata Corporation, College Station, Texas, USA).

## Results

**Figure 4:**
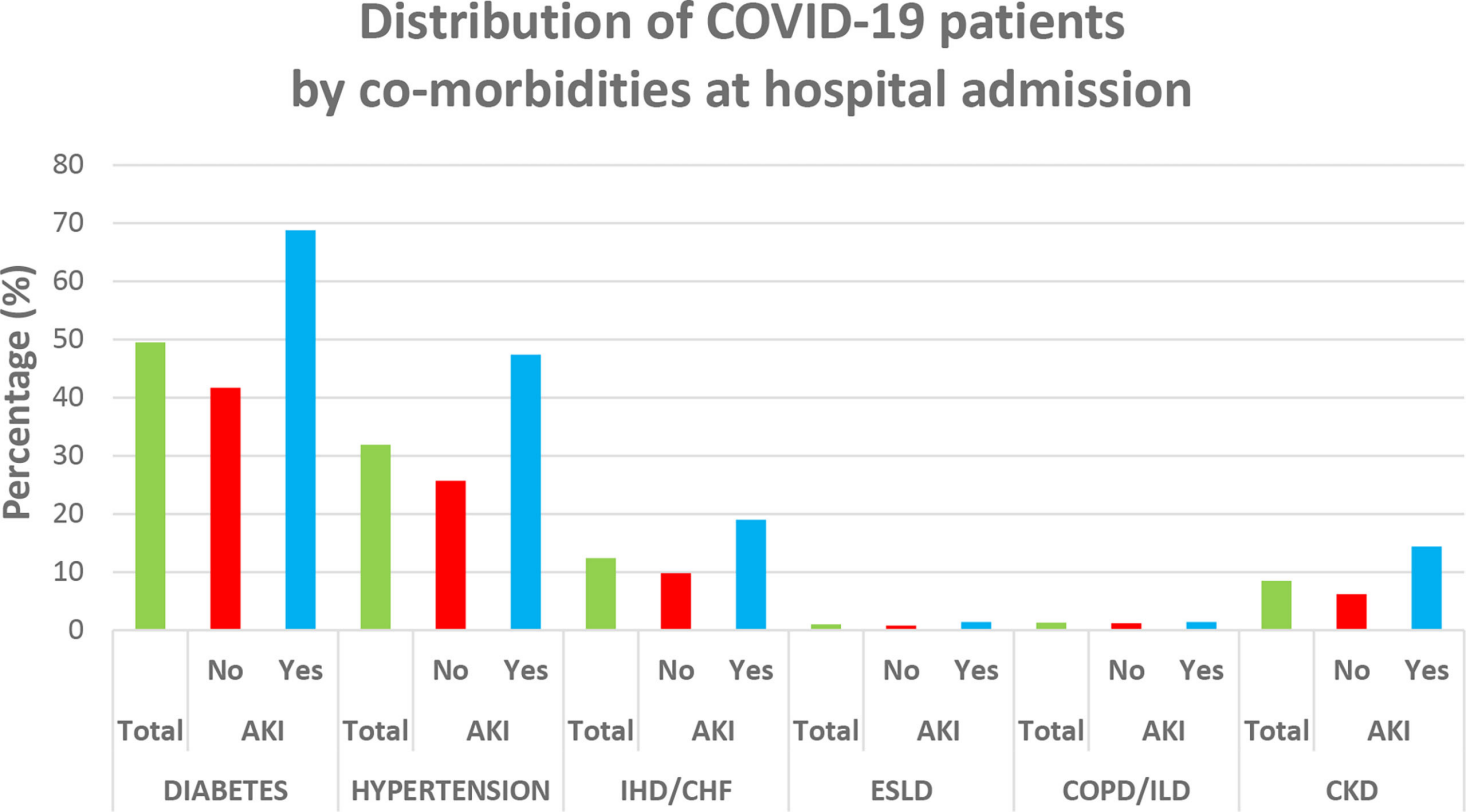
Distribution of comorbidities among COVID-19 patients at hospital admission. IHD, ischemic heart disease; CHF, chronic heart failure; CKD, chronic kidney disease; COPD, chronic obstructive pulmonary disease; ILD, interstitial lung disease; ESLD, end-stage liver disease.

As shown in **Table 1**, 37.3% patients were affected by severe COVID-19, whose average age was 56.6 ± 14.7 years, with 60% (N = 599) of them being males. The mean LoS was 8.8 days, 33% (=330/997) had to be admitted to ICU, and 19% (=192/997) died. AKI was more prevalent in male patients and increased with age and severity of COVID-19 and among those referred to ICU, with a progressively higher prevalence with increasing number of days between hospital admission and ICU referral. The most common comorbidity was diabetes mellitus (49.5% = 493/997) followed by hypertension (31.9% = 318/997), ischemic heart disease and heart failure (12.4% = 124/997), and chronic kidney disease (8.5% = 85/997). With the exception of COPD and liver disorders, all pre-existing morbidities were significantly higher among patients developing AKI (**Figure 4**). Finally, patients developing AKI were featured by higher levels of BUN, creatinine, WBC, and LDH (**Table 1**).

**Table 1:** Distribution of COVID-19 patients by Acute Kidney Injuries (AKI). Number (N), column percentage (%) and p-value of ANOVA (mean differences) or chi square test (difference in proportions).

FACTORS	STRATA		Total (N=997)N (%)	AKI - N (row %)		p-value
				NO (N=712; 71.5%)	YES(N=285; 28.5%)	
**COVID-19**	**Mild/Moderate**		625 (62.7)	483 (77.3)	142 (22.7)	<0.001
	**Severe**		372 (37.3)	229 (61.6)	143 (38.4)	
**Oxygen** **supplement**	**Mask**		268 (26.9)	241 (89.9)	27 (10.1)	<0.001
	**Mask with bag reserve**		356 (35.7)	290 (81.6)	66 (18.5)	
	**Non-invasive ventilation**		141 (14.1)	85 (60.3)	56 (39.7)	
	**Intubation**		232 (23.4)	96 (41.4)	136 (58.6)	
**AKI** **Stage**	**Stage 1**		176 (61.8)	–	176	–
	**Stage 2**		59 (20.7)	–	59	
	**Stage 3**		50 (17.5)	–	50	
**Timeline of** **AKI onset** (days since admission)	**Mean ± SD**		5.6 ± 3.5	–	–	–
	**<2**		46 (16.1)	–	46	–
	**2-3**		55 (19.3)	–	55	
	**4-6**		96 (33.7)	–	96	
	**7+**		88 (30.9)	–	88	
**LoS** **(days)**	**Mean ± SD**		8.80 ± 4.35	7.84 ± 3.35	11.19 ± 5.52	<0.001
	**<7**		322 (32.3)	270 (83.9)	52 (16.2)	<0.001
	**7-9**		369 (37.0)	286 (77.5	83 (22.5)	
	**10-13**		175 (17.6)	109 (62.3)	66 37.7)	
	**14+**		131 (13.1)	47 (35.9)	84 (64.1)	
**Age** (years)	**Mean ± SD**		56.6 ± 14.7	55.0 ± 14.7	60.8 ± 13.9	<0.001^a^
	**<47**		246 (24.7)	204 (82.9)	42 (17.1)	<0.001
	**47-56**		241 (24.2)	181 (75.1)	60 (24.9)	
	**57-65**		249 (25.0)	166 (66.7)	83 (33.3)	
	**66+**		261 (26.2)	161 (61.7)	100 (38.3)	
**Sex**	**Female**		398 (39.9)	308 (77.4)	90 (22.6)	0.001
	**Male**		599 (60.1)	404 (67.5)	195 (32.6)	
**ICU Admission**	**No**		667 (66.9)	532 (79.8)	135 (20.2)	<0.001
	**Yes**		330 (33.1)	180 (54.6)	150 (45.5)	
**Timeline of** **ICU** (days since admission)	**Mean ± SD**		3.7 ± 2.5	3.6 ± 2.3	3.9 ± 2.6	0.252
	**<2**		127 (38.5)	73 (57.5)	54 (42.5)	0.277
	**3-5**		120 (36.4)	68 (56.7)	52 (43.3)	
	**6+**		83 (25.2)	39 (47.0)	44 (53.0)	
**Mortality**	**No**		805 (80.7)	650 (80.8)	155 (519.3)	<0.001
	**yes**		192 (19.3)	62 (32.3)	130 (67.7)	
**Comorbidities**	**Diabetes mellitus**	**No**	504 (50.6)	415 (82.3)	89 (17.7)	<0.001
		**Yes**	493 (49.5)	297 (60.2)	196 (39.8)	
	**Hypertension**	**No**	679 (68.1)	529 (77.9)	150 (22.1)	<0.001
		**Yes**	318 (31.9)	183 (57.6)	135 (42.5)	
	**IHD, CHF**	**No**	873 (87.6)	642 (73.5)	231 (26.5)	<0.001
		**Yes**	124 (12.4)	70 (56.5)	54 (43.6)	
	**ESLD**	**No**	987 (99.0)	706 (71.5)	281 (28.5)	0.422 ^b^
		**Yes**	10 (1.0)	6 (60.0)	4 (40.0)	
	**COPD-ILD**	**No**	984 (98.7)	703 (71.4)	281 (28.6)	0.861 ^b^
		**Yes**	13 (1.3 )	9 69.2)	4 (30.8)	
	**CKD** (eGFRr<45)	**No**	912 (91.5)	668 (73.3)	244 (26.8)	<0.001
		**Yes**	85 (8.5 )	44 (51.8)	41 (48.2)	
**Electrolyte** **imbalance**	**Na^+^** (mEq/L)	**Mean ± SD**	134.91 ± 4.95	135.21 ± 4.69	134.18 ± 5.49	0.004
		<135	435 (44.9)	286 (65.8)	149 (34.3)	<0.001
		135-145	517 (53.4)	396 (76.6)	121 (23.4)	
		>145	16 (1.5)	8 (50.0)	8 (50.0)	
	**K^+^** (mEq/L)	**Mean ± SD**	4.17 ± 0.60	4.13 ± 0.53	4.28 ± 0.74	<0.001
		<3.5	79 (8.2)	54 (68.4)	25 (31.7)	<0.001
		3.5-5.0	812 (84.0)	598 (73.7)	214 (26.4)	
		> 5	76 (7.9)	36 (47.4)	40 (52.6)	
	**Mg ^2+^** (md/dL)	**Mean ± SD**	1.89 ± 0.39	1.90 ± 0.36	1.87 ± 0.46	0.370^a^
		<1.7	214 (26.0)	244 (67.4)	118 (32.6)	0.008
		1.7-3	684 (73.4)	444 (73.5)	160 (26.5)	
		>3	5 (0.6)	1 (20.0)	4 (80.0)	
	**Ca ^2+^** (mg/dL)	**Mean ± SD**	8.81 ± 0.97	8.86 ± 0.92	8.70 ± 1.09	0.080
		<8.8	179 (32.2)	117 (63.6)	62 (34.6)	0.047
		8.8-10.5	367 (66.0)	276 (75.2)	91 (24.0)
		>10.5	10 (1.8)	8 (80.0)	2 (20.0)
**eGFR** (ml/min/1.73m^2^)		**Mean ± SD**	78.35 ± 26.46	79.71 ± 25.49	74.96 ± 28.52	0.010
		<90	698 (70.0)	248 (24.1)	51 (5.1)	0.934
		90-119	248 (24.9)	175 (70.6)	73 (29.4)	
		120+	51 (5.1)	3 (72.6)	14 (27.5)	
**WBC** (× 109/L)		**Mean ± SD**	8.73 ± 7.19	8.16 ± 6.53	10.15 ± 9.08	<0.001
		<5.2	238 (23.1)	183 (76.9)	55 (23.1)	<0.001
		5.2-7.1	255 (25.6)	193 (75.7)	62 (24.3)	
		7.2-10.4	253 (25.4)	182 (71.9)	71 (28.1)	
		10.5+	252 (25.2)	154 (61.4)	97 (38.7)	
**Platelets** (× 109/L)		**Mean ± SD**	209.72 ± 93.93	214.25 ± 93.08	198.38 ± 95.26	0.016
		<147	249 (25.0)	169 (67.9)	80 (32.1)	0.333
		147/190	249 (25.0)	175 (70.3)	74 (29.7)	
		191/260	247 (24.8)	179 (72.5)	68 (275)	
		261+	252 (25.3)	189 (75.0)	63 (25.0)	
**LDH** (U/L)		**Mean ± SD**	848.60 ± 619.23	765.63 ± 345.35	1058.57 ± 996.71	<0.001
		<564	216 (25.0)	1771 (81.9)	39 (18.1)	<0.001
		564/753	213 (24.6)	166 (77.9)	47 (22.1)	
		754-997	216 (25.0)	150 (69.4)	66 (30.6)	
		978+	220 (25.4)	127 (57.7)	93 (42.3)	
**BUN** (mg/dL)		**Mean ± SD**	19.23 ± 10.51	17.41 ± 9.30	23.80 ± 11.91	<0.001
		<14	240 (24.1)	211 (87.9)	29 (12.1)	<0.001
		14-15	172 (17.3)	135 (78.5)	37 (21.5)	
		16-20	316 (317)	228 (72.2)	88 (27.9)	
		21+	269 (26.9)	138 (51.3)	131 (48.7)	
**Blood Creatinine** (mg/dL)		**Mean ± SD**	1.14 ± 0.74	1.08 ± 0.74	1.27 ± 0.74	<0.001
		<0.7	17 (1.7)	15 (88.2)	2 (11.8)	<0.001
		0.7-1.2	749 (75.1)	584 (78.0)	165 (22.0)	
		>1.2	231 (232)	113 (48.9)	118 (51.1)	
**Hemoglobin (g/dl)**		**Mean ± SD**	13.55 ± 2.10	13.69 ± 1.95	13.21 ± 2.4	0.001

LoS, Length of hospital stay; ICU, Intensive care unit; AKI, Acute kidney injury; IHD, Ischemic heart disease; CHF, Chronic heart failure; CKD, Chronic kidney disease; COPD, chronic obstructive pulmonary disease; ILD, Interstitial lung disease; ESLD, End stage liver disease; WBC, White Blood Cells; LDH, Lactate de-hydrogenase; eGFR, Estimated Glomerular Filtration Rate.

As is shown in **Table 2**, the majority of patients were referred to ICU within 2 days (N = 127, 38.5%) or 3–5 days (N = 120; 36.4%) since hospital admission, and the increasing timeline between hospitalization and ICU referral translated into longer LoS. Prevalence of AKI was higher among patients affected by a milder form of COVID-19 referred to the ICU within 2 days since hospital admission, whereas among those developing AKI 3+ days since admission to hospital, it progressively increased with days since hospital admission to ICU referral (42.5% for patients admitted <2 days since hospital admission to 53.0% among those referred to the ICU 6+ days since hospital admission). The death rate was 19.3% (=192/997), 67.7% (=130/192) vs. 32.3% (=62/192) among those not developing AKI (**Table 2**). The majority of COVID-19 patients developed AKI after ICU referral, and the death rate also increased with days since hospital admission to ICU referral (**Table 2**).

**Table 2:** Distribution of timeline of intensive care unit (ICU) admission, by severity of COVID-19, length of hospital stay (LoS, in days), acute kidney injury (AKI) onset (yes vs. no), and patient outcome (death vs. survival). Number (N), percentages (%), mean (M) ± standard deviation (SD).

ICU admission			LoS (days)	AKI onset Number (row %)					Patient outcome N (row %)			
Timeline (days since hospital admission)	Patients’ breakdown	N (%)	M ± SD	No N = 712 (71.5)	Yes N = 285 (28.5)	Before ICU admission(N = 30)	Same day as ICU admission(N = 22)	After ICU admission (N = 98)	Survived		Deceased	
									All patients N = 805 (80.7)	AKIN = 155 (19.3)	All patients N = 192 (19.3)	AKI N = 130 (45.6)
**<2**	**All**	127 (38.5)	9.5 ± 5.9	73 (57.5)	54 (42.5)	5 (9.3)	7 (13.0)	42 (77.8)	72 (56.7)	15 (27.8)	55 (43.3)	39 (72.2)
	**Mild/moderate COVID-19**	47 (37.0)	8.9 ± 4.9	29 (61.7)	18 (30.3)	4 (22.2)	4 (22.2)	10 (55.6)	30 (63.8)	8 (44.4)	17 (36.2)	10 (55.6)
	**Severe COVID-19**	80 (63.0)	9.8 ± 6.4	44 (55.4)	36 (45.0)	1 (2.8)	3 (8.3)	32 (88.9)	42(52.5)	29 (80.6)	38 (47.5)	7 (19.4)
**3–5**	**All**	120 (36.4)	11.0 ± 5.1	68 (56.7)	52 (43.3)	13 (25.0)	7 (13.5)	32 (61.5)	53 (44.2)	6 (11.5)	67 (55.8)	46 (88.5)
	**Mild/moderate COVID-19**	58 (48.3)	10.6 ± 5.4	37 (65.8)	21 (36.2)	6 (28.6)	3 (14.3)	12 (57.1)	29 (50.0)	4 (19.1)	29 (50.0)	17 (81.0)
	**Severe COVID-19**	62 (51.7)	11.5 ± 4.9	31 (50.0)	31 (50.0)	7 (22.6)	4 (12.9)	20 (64.5)	24 (38.7)	2 (6.5)	38 (61.3)	29 (93.4)
**6+**	**All**	83 (25.2)	13.1 ± 4.7	39 (47.0)	44 (53.0)	12 (27.3)	8 (18.2)	24 (54.6)	27 (32.5)	11 (25.0)	56 (76.5)	13 (75.0)
	**Mild/moderate COVID-19**	30 (36.1)	13.7 ± 5.2	16 (53.3)	14 (46.7)	1 (7.1)	5 (35.7)	8 (57.1)	12 (40.0)	6 (42.9)	18 (60.0)	8 (57.1)
	**Severe COVID-19**	53 (63.9)	12.7 ± 4.4	23 (43.4)	30 (56.6)	11 (36.7)	3 (10.0)	16 (53.3)	15 (28.3)	25 (83.3)	38 (71.7)	5 (16.7)

As is shown in **Table 3**, both BUN and blood creatinine increased considerably more for COVID-19 patients developing AKI, from admission to end of follow-up.

**Table 3:** Variation of blood urea nitrogen (BUN) and blood creatinine from hospital admission to end of follow-up (hospital discharge or death).

CLINICAL PARAMETERS		Patients without AKI				Patients developing AKI			
		Admission	Final	Difference	p-value	Admission	Final	Difference	p-value
**BUN** (mg/dL)	M ± SD	17.41 ± 9.30	19.02 ± 10.62	-1.6 1± 4.49	<0.001	23.8 ± 11.9	40.5 ± 24.2	16.72 ± 21.28	<0.001
	Median (IQR)	16 (13; 19)	17 (13; 21)	0 (-3; 0)		20 (16; 28)	31 (23; 50)	-10 (24; -4)	
**Blood Creatinine** (mg/dL)	M ± SD	1.08 ± 0.74	1.11 ± 0.76	-0.03 ± 0.16	<0.001	1.27 ± 0.74	2.09 ± 1.53	0.82 ± 1.23	<0.001
	Median (IQR)	1 (0.9; 1.1)	1 (0.9; 1.1	0 (-0.1; 0)		1 (0.9; 1.4)	1.5 (1.3; 2.2)	-0.4 (-0.9;-0.3)	

MEAN (M) ± standard deviation (SD). Difference, difference between baseline (hospital admission) to end of follow-up (final); Wilcoxon test p-value; IQR, interquartile range (IQR). AKI, acute kidney injury; BUN, blood urea nitrogen.

**Table 4** displays the results of a multiple Cox regression model on AKI onset among study subjects hospitalized for COVID-19. COVID-19 patients who at hospital admission were affected by diabetes mellitus (HR = 1.72; 95% CI: 1.26; 2.38), hyperkalemia (HR = 1.64; 95% CI: 1.08; 2.48), progressively higher levels of BUN (>14 mg/dl), and WBC count (>5.1 × 10^9^/L) were more likely to develop AKI during hospitalization.

**Table 4:** Multiple Cox regression model on the risk of acute kidney injuries (AKI).

Factors	Strata	Univariable analysis HR (95% CI)	Multivariable analysis aHR (95% CI)
**Diabetes mellitus**	**No**	Reference	Reference
	**Yes**	1.70 (1.32; 2.19)	1.69 (1.24; 2.33)
**Any other** comorbidity*	**No**	reference	
	**Yes**	1.43 (1.13; 1.83)	
**Na^+^** (mEq/L)	**<135**	1.36 (1.07; 1.73)	
	**135–145**	Reference	
	**>135**	1.56 (0.76; 3.20)	
**K^+^** (mEq/L)	**<3.5**	1.25 (0.82; 1.89)	1.16 (0.73; 1.85)
	**3.5–5**	Reference	Reference
	**>5**	1.95 (1.39; 2.74)	1.63 (1.08; 2.47)
**Mg^2+^** (mg/dL)	**<1.7**	0.85 (0.63; 1.15)	
	**1.7–3.0**	Reference	
	**>3.0**	3.86 (1.43; 10.47)	
**WBC** (× 10^9^/L)	**<5.2**	Reference	Reference
	**5.2–7.1**	1.45 (1.01; 2.10)	1.53 (1.02; 2.31)
	**7.2–10.4**	1.42 (1.00; 2.02)	1.43 (0.95; 2.15)
	**10.5+**	1.80 (1.29; 2.51)	1.52 (1.03; 2.25)
**BUN** (mg/dL)	**<14**	Reference	Reference
	**14–15**	1.73 (1.06; 2.83)	1.98 (1.12; 3.48)
	**16–20**	2.33 (1.52; 3.57)	2.44 (1.49; 4.01)
	**21+**	2.26 (1.49; 3.41)	2.29 (1.43; 3.67)
**eGFR** (ml/min/1.73 m^2^)	**<90**	0.77 (0.59; 1.00)	
	**90–120**	Reference	
	**120+**	1.41 (0.79; 2.51)	

*Hypertension; ischemic heart disease: chronic heart failure; chronic kidney disease; chronic obstructive pulmonary disease; interstitial lung disease; end-stage liver disease. Hazard ratio unadjusted (HR) and adjusted (aHR) with 95% confidence interval (95%CI). Multiple regression model fitted on 814 complete (case analysis) observations and adjusted for diabetes mellitus, any comorbidity, natremia, kalemia, magnesemia, WBC,white blood cells (WBCs) and blodd urea nitrogen (BUN) at admission.

**Table 5** displays the results of a multivariable Cox regression model for in-hospital mortality. The untoward prognostic risks were found among patients who at admission were affected by hypokalemia (HR = 2.23; 95% CI: 1.32; 3.78), higher levels of BUN (>16 mg/dl), and LDH and increasing WBC count (>7.2 × 10^9^/l). At multivariable analysis, ICU admission significantly increased the risk of death from severe COVID-19 (HR = 7.34; 95% CI: 2.41; 22.35) and even more from mild/moderate COVID-19 (HR = 10.14; 95% CI: 3.55; 28.98). No other interactions (between ICU and AKI, and between COVID-19 and AKI) were statistically significant.

**Table 5:** Multiple Cox regression model on the risk of death.

Factors	Strata	Univariable analysis HR (95%CI)	Multivariable analysis aHR (95%CI)
**Age**	**<47**	Reference	
	**47–56**	1.66 (0.93; 2.97)	
	**57–65**	2.03 (1.16; 3.56)	
	**66+**	2.27 (1.32; 3.90)	
**AKI**	**No**	Reference	
	**Yes**	1.91 (1.39; 2.63)	
**COVID-19**	**Mild/Moderate**	Reference	
	**Severe**	1.72 (1.29; 2.31)	
**ICU ** **admission**	**No**	Reference	
	**Yes**	10.27 (5.90; 17.87)	
**Interaction** **ICU admission** **X** **COVID-19 severity**	**Mild/moderate COVID-19 and no ICU admission**	Reference	Reference
	**Severe COVID-19 and no ICU admission**	1.28 (0.43; 3.81)	1.07 (0.25; 4.56)
	**Mild/moderate COVID-19 and ICU admission**	10.22 (5.05; 20.71)	10.14 (3.55; 28.98)
	**Severe COVID-19 and ICU admission**	11.72 (5.89; 23.31)	7.34 (2.41; 22.35)
**Diabetes mellitus**	**No**	Reference	
	**Yes**	2.26 (1.62; 3.16)	
**Any** comorbidity*	**No**	Reference	
	**Yes**	1.81 (1.33; 2.46)	
**Na^+^** (mEq/L)	**<145**	1.19 (0.89; 1.61)	
	**135–145**	Reference	
	**>145**	2.69 (1.39; 5.19)	
**K^+^** (mEq/L)	**<3.5**	2.17 (1.43; 3.30)	2.23 (1.32; 3.78)
	**3.5–5.0**	Reference	Reference
	**>5.0**	1.88 (1.22; 2.88)	1.46 (0.80; 2.66)
**Mg^2+^** (mg/dL)	**<1.7**	0.90 (0.63; 1.29)	
	**1.7–3**	Reference	
	**>3**	7.05 (2.57; 19.34)	
**Platelets** (× 10^9^/L)	**<147**	Reference	Reference
	**147/190**	0.67 (0.46; 0.99)	0.80 (0.47; 1.39)
	**191/260**	0.68 (0.47; 1.00)	0.56 (0.33; 0.94)
	**261+**	0.84 (0.56; 1.27)	0.70 (0.41; 1.17)
**BUN** (mg/dL)	**<14**	Reference	Reference
	**14–15**	1.35 (0.70; 2.60)	1.91 (0.86; 4.25)
	**16–20**	2.55 (1.51; 4.33)	2.31 (1.19; 4.46)
	**21+**	2.33 (1.40; 3.87)	1.51 (0.78; 2.91)
**LDH** (U/L)	**<564**	Reference	Reference
	**564/753**	1.03 (0.55; 1.92)	1.44 (0.69; 3.01)
	**754–997**	1.64 (0.93; 2.89)	1.63 (0.77; 3.48)
	**978+**	2.59 (1.53; 4.38)	2.53 (1.13; 4.69)
**WBC** (× 10^9^/L)	**<5.2**	Reference	Reference
	**5.2–7.1**	1,34 (0.83; 2.16)	1.75 (0.93; 3.26)
	**7.2–10.4**	1.53 (0.98; 2.38)	1.88 (1.04; 3.40)
	**10.5+**	2.30 (1.53; 3.45)	2.51 (1.41; 4.46)

Hazard ratio (HR) with 95% confidence interval (95% CI). Model fitted on 755 complete (case analysis) observations and adjusted for severity of COVID-19, AKI, diabetes, any comorbidity. *Natremia, kalemia, magnesemia, WBC, platelets, LDH, and BUN at admission.

*Hypertension; ischemic heart disease: chronic heart failure; chronic kidney disease; chronic obstructive pulmonary disease; interstitial lung disease; end-stage liver disease Hazard Ratio unadjusted (HR) and adjusted (aHR) with 95 confidence interval (95%CI). AKI, acute kidney injury; ICU, Intensive care unit; BUN, blood urea nitrogen; LDH, lactate dehydrogenase; WBC, white blood cells.

## Discussion

### Key Findings

The prevalence of AKI in the present study was 28.5%, a figure fairly in line with reports from different settings and time periods (32). The risk of AKI increased with diabetes mellitus as well as hyperkalemia, higher WBC count, and increasing level of BUN measured at hospital admission. On the other hand, the overall mortality risk among COVID-19 patients was 19.3%. Factors associated with a higher risk of death were ICU admission for severe COVID-19 as well as for mild/moderate COVID-19, hypokalemia, higher level of BUN, increasing WBC, and increasing LDH measured at hospital admission.

### Interpretation of Findings

In a systematic review and meta-analysis on 14,415 COVID-19 patients from different countries, the prevalence of AKI was 11% (95% CI: 0.07–0.15; p < 0.01), hence a figure much lower than the present study. Moreover, in the latter meta-analysis AKI was significantly associated with death (OR = 8.45; 95% CI: 5.56–12.56; p < 0.001) and severe COVID-19 (OR = 13.52; 95% CI: 5.43–33.67; p < 0.001) among hospitalized patients (33). In our prospective study—where a survival analysis by Cox proportional-hazard models was employed to examine risk factors influencing AKI incidence and mortality risk in a particular point in time during hospitalization—although 28.5% COVID-19 patients developed AKI and the crude death rate was higher among patients developing it (67.7%), AKI did not increase the adjusted mortality risk at multivariable analysis, a rather unexpected finding. The majority of COVID-19 patients developed AKI after ICU referral; therefore, the risk of death appeared to rise following ICU admission rather than AKI. Almost 40% patients were referred to the ICU within 2 days since hospital admission, suggesting critical clinical conditions of these patients even with less severe form of COVID-19.

**Figure 5:**
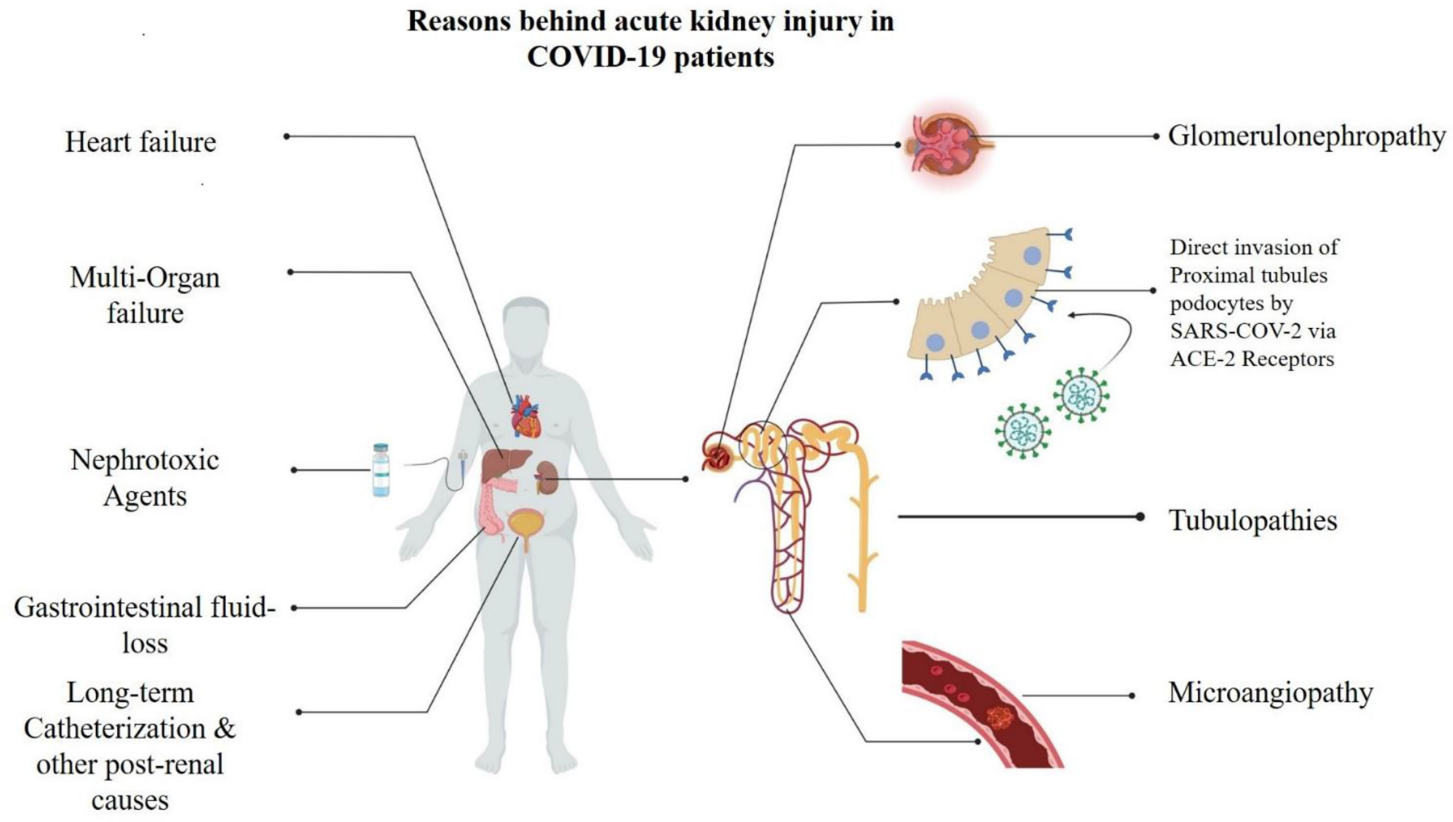
Underlying mechanims contributing to onset of acute kidney injuries (AKI) in COVID-19 patients.

Kidney involvement and AKI onset in COVID-19 patients have multiple risk factors, and several explanatory mechanisms have been proposed (**Figure 5**), including electrolyte imbalance, medication-induced injury, organ failure in late stages of the disease, impairment of gas exchange, hemodynamic alterations including right heart failure, systemic congestion due to fluid overload, and secondary infections/sepsis, among others (32, 34).

AKI is reportedly a frequent pattern in patients dying from COVID-19 (15, 35–38), with acute tubular injury being the most common morpho-pathologic finding in kidney autopsies (37–39). Furthermore, ACE-2 and TMPRSS2 are highly expressed in proximal tubules, where SARS-CoV-2 particles could be detected postmortem in the respective podocytes from COVID-19 patients (40), hinting that the novel coronavirus can directly target the kidneys (7, 10, 34, 41). Experimental and epidemiological studies evidenced that SARS-CoV-2 can infect and damage target renal epithelial cells expressing ACE-2 and TMPRSS2, triggering a cytokine storm (sustained especially by IL-6 and interferon) and directly causing AKI by increasing vascular permeability, shock, and multiorgan failure or aggravating/perpetuating a kidney injury already initiated by non-viral processes (37, 38, 42). Less frequent autoptic findings include micro-angiopathy and collapsing glomerulopathy (40), a frequent cause of proteinuria rapidly progressing to kidney failure (43–45), which predominantly affect patients with homozygous apolipoprotein L1 (APOL-1) high-risk alleles (45–46).

This single-center clinical study was conducted from October 22, 2020, to January 7, 2021, during the third pandemic wave in Iran (**Figure 1**), before large-scale vaccination campaigns against COVID-19 were deployed globally. The massive surge of COVID-19 cases did not allow to perform autopsies or biopsies on patients with AKI.

Although SARS-CoV-1 proved capable of infecting kidney cells *in vitro* (47, 48), the evidence supporting persistent infection of the kidney by SARS-CoV-2 is still unconvincing (45). An alternative plausible pathogenetic hypothesis is the “hit and run” model, where the renal injury persists after the clearance of an early direct kidney infection by SARS-CoV-2. However, AKI associated with COVID-19 is probably determined by multiple factors, including an indirect organ damage induced by the physiologic disturbances caused by COVID-19 and the therapies administered to treat the severe acute respiratory syndrome (9, 32, 34, 45). Contrast media, corticosteroids, NSAIDs, ACEs, Angiotensin receptor blockers (ARBs), and various antibiotics are reportedly associated with increased risk of AKI in COVID-19 patients (34, 40), although there is also some evidence that high daily doses (40 mg) of methylprednisolone are associated with increased mortality but lower risk of AKI in COVID-19 patients (49). In a meta-analysis on 23,655 hospitalized, critically ill COVID-19 patients, the incidence of AKI was not significantly different between COVID-19 patients (51%) and critically ill patients infected with ACE2-associated (56%) or non-ACE2-associated viruses (63%). The latter meta-analysis estimated a lower risk of renal replacement therapy in patients affected by COVID-19 or ACE2-associated viruses (featured by a lower risk of shock and use of vasopressors) as compared with patients infected with non-ACE2-binding viruses (50).

As already mentioned, considerable inconsistencies exist regarding the prevalence of AKI in hospitalized COVID-19 patients, with figures widely ranging from 0.5% to 80% (14). Explanatory factors for the inconsistency of the epidemiological evidence on AKI prevalence among COVID-19 patients include ethnicity, genetic polymorphism, type of SARS-CoV-2 variant, the underlying mechanism of kidney injury (either pre-renal, renal, or post-renal), and the methodology employed in various studies (19, 41, 45, 51).

Up until November 14, 2021 (before the spread of the Omicron variant), 56,900,000 cumulative COVID-19 infections were recorded in Iran, where the overall infection/hospitalization rate was 1%–3% and the mortality rate equaled to 277.5/100,000 (11). According to a systematic review and meta-analysis, in Iran the proportion of hospitalized COVID-19 patients developing AKI was 24% (95% CI: 17%–31%), slightly lower than figures from the present study (28.5%) (52). In an earlier study conducted in Iran during February–April 2020, the prevalence of AKI was lower (13.8%) [53]. A lower prevalence of AKI reported from China in the early stages of the COVID-19 pandemic could also be due to underestimation of signs and symptoms not involving the respiratory system (16, 17). Moreover, clinical management of COVID-19 has considerably evolved over time since the early days of the pandemic, and this may account for the inconsistency in AKI figures reported by different studies in diverse periods.

Despite the inconsistencies of prevalence among COVID-19 patients, common risk factors of AKI according to the open literature are advanced age, male gender, and comorbidities such as diabetes mellitus, hypertension, CKD, ischemic heart disease, electrolyte imbalance, and inflammatory markers (22, 23, 32, 36, 41, 54). Likewise, the role of diabetes, electrolyte imbalance, and inflammation in AKI was confirmed in the present study. These factors were already present at hospital admission, reflecting critical clinical conditions of patients entering the hospital independently from the severity of their viral lung disease, thereby supporting the hypothesis that treatment and alterations induced also by mild/moderate forms of COVID-19 may contribute to MOF, including AKI. The enhanced risk of AKI in males may reflect on one side a higher SARS-CoV-2 infection rate in males, on the other side their higher susceptibility to viral infections due to differences in natural immunity linked to sex chromosomes (41). The enhanced expression of ACE2 and TMPRSS2 receptors in males, regulated by androgens, might account for their higher susceptibility to severe COVID-19 (41, 55, 56). In contrast, estrogen may inhibit the cell invasion of SARS-CoV-2 by reducing the expression of TMPRSS2 (57).

## Strengths and Limitations

This study has several strengths, including a high number of hospitalized COVID-19 patients and a detailed and thorough collection of clinical variables with a good level of completeness of data, allowing to adjust risk estimates. Furthermore, rather than a cross-sectional design, this study employs a longitudinal approach and it is the first to test the interaction between ICU and severity of COVID-19, thereby disentangling the impact of ICU referral by COVID-19 severity on the mortality risk. Nevertheless, this study was conducted in the midst of the third pandemic wave in Iran, with no capacity to perform postmortem autopsies in AKI patients. Since autopsy and biopsy are essential steps to elucidate the exact mechanism of AKI in COVID-19 patients, future studies should include postmortem examination in COVID-19 patients affected by AKI.

## Conclusions

The prevalence of AKI—a relatively common finding among hospitalized COVID-19 patients—was 28.5% in the present study, and the overall mortality rate was 19.3%. The risk of AKI was associated with diabetes mellitus hyperkalemia, electrolyte imbalance, and inflammation, but not with the severity of COVID-19. However, AKI did not influence the mortality risk, which increased with joint association between ICU admission and severe COVID-19 as well as mild/moderate COVID-19, hypokalemia, and higher levels of BUN, WBC, and LDH measured at hospital admission. Diabetes mellitus and comorbidities did not increase the mortality risk among these hospitalized COVID-19 patients. AKI can occur anytime in the course of COVID-19 as a possible complication arising from disturbances and therapies administered to treat even milder forms of the disease. Therefore, considering the crucial importance played by the kidneys in regulating blood pressure and filtering blood from toxic substances, COVID-19 patients should be early monitored for the onset of eventual complications, as they may be already in critical clinical condition at hospital admission.

Future research should focus on biomarkers of tubule damage predicting AKI and whether modulation of ACE2 expression by renin-angiotensin system inhibitors may be beneficial for COVID-19 patients, diminishing the risk of AKI. Finally, since the underlying cause of AKI is invasion of kidney cells by SARS-CoV-2, research should focus on drugs capable of interfering with the binding of SARS-CoV-2 with cell receptors (ACE2 and TMPRSS2), with cell endocytosis of the virus and altering the pH of lysosomes where the virus crosses once inside the cell.

## Data Availability Statement

The raw data supporting the conclusions of this article will be made available by the authors, without undue reservation.

## Ethics Statement 

The study was approved by the Medical Ethical Committee of Baqiyatallah University of Medical Sciences. Written informed consent for participation was not required for this study in accordance with the national legislation and the institutional requirements.

## Author Contributions

ZR, BE, EN, SSH, ME, MJ, SHS, MA, ZE, and BHB designed the study etc. designed the study, collected the clinical data and samples, drafted the primary version of the manuscript, and validated the final draft. GM and LC analyzed/interpreted the data and wrote the manuscript. The authors approved the final manuscript.

## Conflict of Interest

The authors declare that the research was conducted in the absence of any commercial or financial relationships that could be construed as a potential conflict of interest.

The reviewer AA declared a shared affiliation with the author SS to the handling editor at the time of review.

## Publisher’s Note

All claims expressed in this article are solely those of the authors and do not necessarily represent those of their affiliated organizations, or those of the publisher, the editors and the reviewers. Any product that may be evaluated in this article, or claim that may be made by its manufacturer, is not guaranteed or endorsed by the publisher.
